# *ORF Ι* of Mycovirus SsNSRV-1 is Associated with Debilitating Symptoms of *Sclerotinia sclerotiorum*

**DOI:** 10.3390/v12040456

**Published:** 2020-04-17

**Authors:** Zhixiao Gao, Junyan Wu, Daohong Jiang, Jiatao Xie, Jiasen Cheng, Yang Lin

**Affiliations:** 1 The Provincial Key Lab of Plant Pathology of Hubei Province, College of Plant Science and Technology, Huazhong Agricultural University, Wuhan 430070, China; gaozhixiao@webmail.hzau.edu.cn (Z.G.); wujixiaoyan@163.com (J.W.); daohongjiang@mail.hzau.edu.cn (D.J.); jiataoxie@mail.hzau.edu.cn (J.X.); jiasencheng@mail.hzau.edu.cn (J.C.); 2 State Key Laboratory of Agricultural Microbiology, Huazhong Agricultural University, Wuhan 430070, China

**Keywords:** *sclerotinia sclerotiorum*, sclerotinia sclerotiorum negative-stranded viruses 1, mycovirus, hypovirulence, transcriptome

## Abstract

We previously identified Sclerotinia sclerotiorum negative-stranded virus 1 (SsNSRV-1), the first (−) ssRNA mycovirus, associated with hypovirulence of its fungal host *Sclerotinia sclerotiorum*. In this study, functional analysis of Open Reading Frame Ι (*ORF Ι*) of SsNSRV-1 was performed. The integration and expression of *ORF Ι* led to defects in hyphal tips, vegetative growth, and virulence of the mutant strains of *S. sclerotiorum*. Further, differentially expressed genes (DEGs) responding to the expression of *ORF Ι* were identified by transcriptome analysis. In all, 686 DEGs consisted of 267 up-regulated genes and 419 down-regulated genes. DEGs reprogramed by ORF Ι were relevant to secretory proteins, pathogenicity, transcription, transmembrane transport, protein biosynthesis, modification, and metabolism. Alternative splicing was also detected in all mutant strains, but not in hypovirulent strain AH98, which was co-infected by SsNSRV-1 and Sclerotinia sclerotiorum hypovirus 1 (SsHV-1). Thus, the integrity of SsNSRV-1 genome may be necessary to protect viral mRNA from splicing and inactivation by the host. Taken together, the results suggested that protein ORF Ι could regulate the transcription, translation, and modification of host genes in order to facilitate viral proliferation and reduce the virulence of the host. Therefore, *ORF Ι* may be a potential gene used for the prevention of *S. sclerotiorum*.

## 1. Introduction

Mycoviruses (or fungal viruses), the viruses that infect fungi, are ubiquitous in all kinds of fungi [1]. Although many mycoviruses are commonly associated with cryptic or latent infections of the fungal host, a few of them are reported to confer hypovirulence or hypervirulence to their host. Of these mycoviruses, hypoviruses are considered as potential biological agents to prevent fungal diseases [2]. In Europe, Cryphonectria parasitica hypovirus 1 (CHV1) has been applied to control chestnut blight disease caused by *Cryphonectria parasitica* [3,4,5]. In addition, Rosellinia necatrix megabirnavirus 1 and Sclerotinia sclerotiorum hypovirulence-associated DNA virus 1 are also demonstrated to have the potential to prevent fungal diseases caused by their hosts [5,6,7].

The first negative-stranded RNA virus isolated from *Sclerotinia sclerotiorum* hypovirulent strain AH98, is identified as Sclerotinia sclerotiorum negative-stranded RNA virus 1 (SsNSRV-1). The infection of SsNSRV-1 leads to debilitating symptoms of the fungal host, including defective growth rate, abnormal colonial morphology, curled hyphal tips and hypovirulence [8]. The full length of SsNSRV-1 genome is 10,002 nt with six non-overlapped genes (*ORF Ι-VI*) which are linearly arranged in the genome. Although the genome and organization of SsNSRV-1 are significantly different from those of currently known *mononegaviruses*, this virus is closely related to viruses in families *Nyamiviridae* and *Bornaviridae*, which infect animals. *ORF II* and *ORF V* encode the nucleoprotein (N) and RNA dependent RNA polymerase (RdRp) (L), respectively. It is still unclear whether there are other viral genes encoding structural proteins due to the extreme fragility of the virus particles [8].

*S. sclerotiorum*, an important phytopathogenic ascomycete worldwide, can infect more than 600 plant species including many important cash crops, such as canola, soybean, and sunflower [9]. Many research data demonstrate that *S. sclerotiorum* hosts a variety mycoviruses from ten families and two unclassified genera [10,11,12,13,14,15,16]. This fungus species has been considered as an ideal model for exploring the fungal response to different kinds of mycoviruses [4,10]. The human (-) ssRNA viruses have been studied in depth [17,18], while the fungal (-) ssRNA virus is still poorly understood. SsNSRV-1, which infects *S. sclerotiorum*, provides ideal sample to study the infection and replication of (−) ssRNA virus in fungus.

Alternative RNA splicing (AS) is a kind of post-transcriptional processing mechanism used in eukaryotes to increase the diversity of proteome and to regulate the expression of proteins in different organs and cell types through the formation of different mRNA isoforms from a primary transcript [19,20,21,22]. The phenomenon is ubiquitous in higher eukaryotes. It is estimated that about 90% of genes in the human genome are able to be alternatively spliced [23,24]. Many viruses can take advantage of host splicesome to edit their own transcripts during the infectious cycle, most of which are animal viruses, complex retroviruses and type A influenza virus [24,25,26,27,28]. Although splicing rarely occurs in plant viruses, several plant DNA viruses from two families *Geminiviridae* and *Caulimoviridae* are reported to perform splicing to express some of their proteins [29,30].

In this study, *ORF I* of SsNSRV-1 was cloned and integrated into the genome of *S. sclerotiorum* strain 1980 to conduct the functional analysis for this viral gene. Further, transcriptome analysis was performed to identify the differentially expressed genes (DEGs) and pathways responding to the expression of *ORF I.* The DEGs associated with secretory proteins and pathogenicity were also investigated to determine whether ORF I could regulate the virulence of *S. Sclerotiorum*. This study will help us to understand the function of viral gene *ORF I* in the infection and proliferation of SsNSRV-1 in its fungal host and may provide new insights towards the molecular mechanisms of virus-induced hypovirulence.

## 2. Materials and Methods 

### 2.1. Fungal Materials and Growth Conditions 

*S. sclerotiorum* wild-type strain 1980 (virus-free) and hypovirulent strain AH98, which was co-infected by two mycoviruses Sclerotinia sclerotiorum negative-stranded RNA virus 1 (SsNSRV-1) and Sclerotinia sclerotiorum hypovirus 1 (SsHV-1), were incubated on potato dextrose agar (PDA) plates at 20 °C. All transgenic strains (Z1-1, Z1-3, Z1-4, Z1-5, Z1-7, Z1-10, Z1-11, Z1-12, Z1-13, Z1-14, and Z1-15), overexpressing *ORF I*, were cultured on PDA plates amended with 100 µg/mL hygromycin B. To collect mycelia for DNA or RNA extraction, wild-type strain 1980, hypovirulent strain AH98 and transgenic strains were incubated on PDA plants overlaid by cellophane membrane at 20 °C. Mycelia were harvested at 2, 4, and 6 days for nucleic acid extraction. Strains AH98 and 1980 were stored on PDA slants at 4 °C. All transgenic strains were stored on PDA slants amended with 100 µg/mL hygromycin B at 4 °C. Before experiments, all strains were cultured on PDA plates for three generations to ensure the stability of their phenotype.

### 2.2. DNA and RNA Extraction, cDNA Synthesis

The fungal mycelia were ground in liquid nitrogen to isolate DNA or RNA. DNA was extracted in CTAB as previously described [31]. Total RNA was isolated using the NI-Sclerotinia sclerotiorum RNA Reagent kit (NEWBIO, Shanghai, China). For transcriptome analysis, mycelial samples of *S. sclerotiorum* hypovirulent strain AH98, virus-free wild-type strain 1980 and two *ORF I* -expressing mutant strains Z1-1 and Z1-13 were harvested at 2, 4, and 6 days post-inoculation (dpi). Equal amount of three samples obtained from different time points were mixed. For each strain, three technical replicates were performed. All RNA samples were stored at −70 °C before use. cDNA was synthesized by the EasyScript^®^ One-Step gDNA Removal and cDNA Synthesis SuperMix (TransGen Biotech, Beijing, China). The concentrations of RNA and DNA were measured by Nanodrop 2000 machine (Thermo Scientific, Waltham, MA, USA™). The quality of them was also verified by agarose-gel electrophoresis.

### 2.3. Molecular Cloning, Vector Construction and Genetic Manipulation

Using first-strand cDNA of strain AH98 as template, the complete cDNA sequences of *ORF I* was amplified by primer pairs (ORF1F/ ORF1R) (Table S1). After gel purification, the amplicon of *ORF I* was cloned into vector pMD19-T (TaKaRa, Dalian, China) and then transformed into competent cells of *Escherichia coli* strain DH5α. The target gene was digested and purified from the plasmid in each positive clone by restriction endonuclease *Kpn* I and then ligated to the expression vector pCH-EF-1 in which *ORF I* was under the control of *EF1α* promoter of *S. sclerotiorum*. After replication in *E. coli*, the binary vectors were transformed into competent cells of *Agrobacterium tumefaciens* strain EHA105. Although strain 1980 and strain AH98 were not isogenic, strain 1980, whose genome was well assembled, was used as the parent strain, due to the lack of virus-free strain of AH98. The expression vector carrying gene *ORF I* was transformed into strain 1980 by Agrobacterium tumefaciens-mediated transformation (ATMT) technique. The insertion fragment of positive clones was verified by sequencing. Two mutants, Z1-1 and Z1-13, were randomly selected from all positive transformants for further study.

### 2.4. Morphological Observation and Pathogenicity Assay

To observe the colonial morphology, all strains were incubated on PDA at 20 °C with 12 h of light and 12 h of dark. After two days incubation, the hyphal tips on PDA were photographed. The diameter of each colony was measured at 24 h post-inoculation (hpi) and 48 hpi, respectively. Vegetative growth rate of each strain was calculated as follows: growth rate (cm/day) = (48 hpi diam. – 24 hpi diam.)/2. The experiment was performed twice with three replicates.

To determine the virulence of *ORF I*-expressing strains, Z1-1 and Z1-13, pathogenicity assay was carried out on detached healthy leaves of canola. Mycelia plugs (diameter = 2.5 mm) from the margins of activated colonies on PDA were placed on detached leaves upside down. The inoculated leaves were incubated in a plastic chamber with 95% of relative humidity at 20 °C. To evaluate disease severity, lesion size was measured 60 h after inoculation. Both wild-type strain 1980 and hypovirulent strain AH98 were used as control. In total, five leaves were inoculated for each strain and three independent replicates were performed.

Statistical analysis of data was performed using ANOVA in the SAS program. Treatment means were compared with the test of significant difference set at the *p* < 0.05 level of confidence according to Duncan’s multiple range test.

### 2.5. Preparation of cDNA Library

cDNA libraries were synthesized to perform transcriptome sequencing for four strains, Z1-1, Z1-13, 1980 and AH98. About 10 mg total RNA of each strain was extracted to construct cDNA library. The quality of total RNA was measured by the QubitFluorometer, Agilent 2100 and NanoDrop, respectively. After treated with DNase I, mRNA was enriched from the total RNA by magnetic beads with Oligo (dT). Then, mRNA was fragmented into about 200 bp segments and purified for end reparation and single nucleotide A (adenine) addition. After that, the short fragments were connected with adapters. The fragments ligated to adapters were selected as templates to synthesize cDNA library. Agilent 2100 Bioanaylzer and ABI StepOnePlus Real-Time PCR System were applied to quantify and qualify the samples. The libraries were sequenced using an Illumina HiSeq 2500 platform (BGI, Shenzhen, China). Transcriptome sequencing was performed for four strains with 3 replicates per each.

### 2.6. Transcriptome Analyses

Via base calling, the original image data were transferred into sequences data and then saved as FASTQ file. To obtain clean reads, the raw reads were filtered to remove adapter-polluted, contaminated, low-quality, and high content of unknown base (N) reads. The clean reads were mapped to the reference sequence of *S. sclerotiorum* strain 1980 UF-70 (Accession: ASM185786v1) using Hisat2 (http://ccb.jhu.edu/software/tophat/index.shtml). The gene expression level was calculated using stringtie (http://cufflinks.cbcb.umd.edu/). Then, differentially expressed genes (DEGs) were extracted with DESeq (http://www.bioconductor.org/packages/release/bioc/html/DESeq.html), using fold change > 2 and *p* value > 0.05 as a screening criterion. Classification of DEGs was based on GO annotation results. GO functional enrichment analysis was conducted using topGO http://www.bioconductor.org/packages/release/bioc/html/topGO.html) [32]. Kyoto encyclopedia of genes and genomes (KEGG) annotation and enrichment analysis were carried out using FungiFun (https://sbi.hki-jena.de/fungifun/fungifun.php) [33].

### 2.7. Real-Time Quantitative Reverse Transcription PCR Analysis

To amplify First-strand cDNA, additional 5 μg of total RNA of each strain, which had been extracted for RNA-Seq, was used as templates. cDNA was synthesized with an oligo d(T) primer by the reverse transcription reagent kit Easy Script^®^for q-PCR. Real-time quantitative reverse transcription PCR (RT-qPCR) was performed using q-PCR SYBR Green mix on a CFX manager system (Bio-Rad, Hercules, CA, USA) in 20 μL reactions. Each reaction consisted of 20 ng of cDNA, 0.5 μL of each primer, and 10 μL master mix. PCR reactions were performed using the thermocycler conditions: 3 min at 95 °C, 40 cycles of 15 s at 95 °C, 15 s at 57 °C, and 20 s at 72 °C. Melting curve analysis of amplification products was performed at the end of each PCR reaction, which verified the presence of a specific product, for 2 min at 16°C °C. The housekeeping gene actin was used as an endogenous control for normalization. The PCR was repeated three times with two replicates per run. Primers used to amplify the target genes were listed in Table S2.

## 3. Results

### 3.1. Predicted Secondary Structure and Function of Protein ORF I

**Figure 1:**
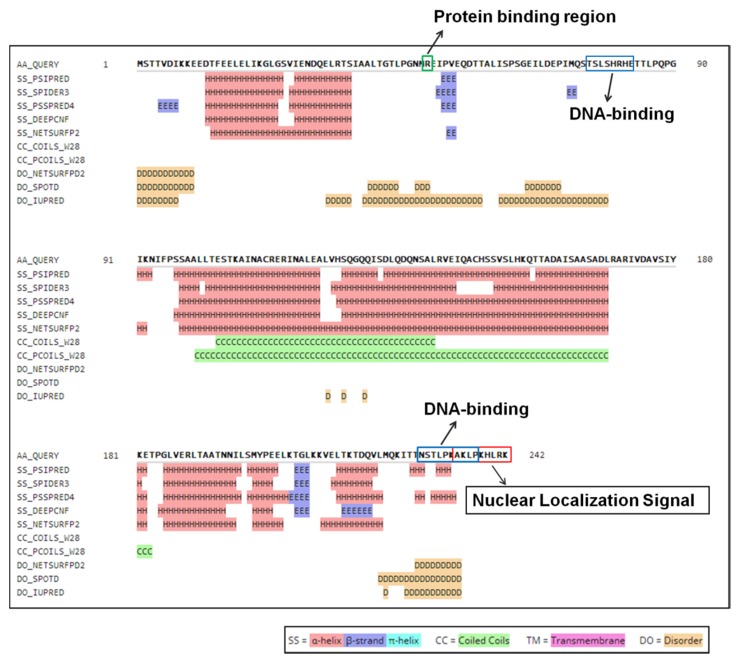
Predicted secondary structure and binding site of protein ORF I. The green box indicates protein binding region. The blue box indicates DNA-binding sites. The red box indicates a nuclear localization signal.

The secondary structure of ORF I was analyzed by bioinformatics software HHpred version 3.2 (https://toolkit.tuebingen.mpg.de/tools/hhpred) [34]. All predicted features are annotated in Figure 1. The results indicated that the secondary structure of ORF I was mainly composed of helical structures, which had two strands and a coiled helix, but no transmembrane helix. There were two DNA-binding sites (76–83, 227–237) and one protein binding region (49) in protein ORF I. All three binding sites were located in the disordered regions. In addition, a nuclear localization signal was predicted at the C-terminal of protein ORF I.

The function of protein ORF I was also predicted by HHpred version 3.2 (https://toolkit.tuebingen.mpg.de/tools/hhpred). The results inferred that ORF I had a high similarity with nucleoporin and might be involved in nucleocytoplasmic transport. In addition, ORF I might also be a transcription factor that could bind to DNA (Table 1). 

The function of viral protein ORF Ⅰ was predicted by HHpred. HHpred, an interactive server for protein homology detection and structure prediction, is very sensitive in finding remote homologs [34], and version 3.2 is used in this study. 

### 3.2. Construction of Expression Vector and Verification of Mutant Strains 

**Figure 2:**
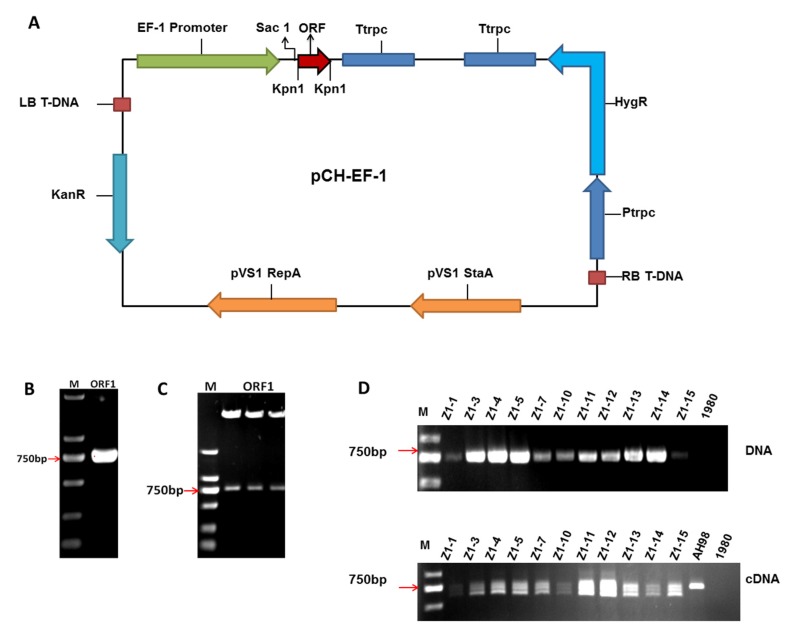
Expression of Open Reading Frame Ι (*ORF I*) of Sclerotinia sclerotiorum negative-stranded RNA virus 1 (SsNSRV-1) in *Sclerotinia sclerotiorum*. (**A**) Schematic diagram of expression vector of *ORF I.* (**B**) The complete sequences of *ORF I* amplified from the cDNA of AH98. (**C**) The insertion of *ORF I* verified by digestion with restriction endonuclease. Overexpression vector of *ORF I* was digested by KpnI to confirm the insertion of *ORF I* in pCH-EF-1. (**D**) The detection of *ORF I* in both transgenic and wild-type strains. Using DNA or cDNA of *ORF I* -expressing transformants (Z1-1, Z1-3, Z1-4, Z1-5, Z1-7, Z1-10, Z1-11, Z1-12, Z1-13, Z1-14 and Z1-15), wild-type strain 1980 and hypovirulent strain AH98 as templates, the sequence of *ORF I* was amplified by primer pair (ORF1F/ORF1R) to screen the transgenic strains. Alternative splicing of *ORF I* mRNA was detected by RT-PCR in all positive transformants but not in strain AH98.

Gene *ORF I* of SsNSRV-1 was amplified from the cDNA of hypovirulent strain AH98 and cloned into vector pMD19-T (Figure 2B). After replication in *E. coli*, the cloning of *ORF I* was confirmed by sequencing. Then the complete gene of *ORF I* was digested by Kpn I and ligated to the linearized vector pCH-EF-1 (Figure 2A). Recombinant expression vector was again digested by KpnI to confirm the insertion of *ORF I*. The size of *ORF I* was verified by gel electrophoresis (Figure 2C). 

To investigate the function of viral gene in fungal host, the expression vector of *ORF I* was transformed into wild-type strain 1980 by ATMT. In all, eleven positive transformants were generated and confirmed by PCR screening with primer pair (ORF1F/ORF1R). When using genomic DNA as templates, only one fragment having the same size with *ORF I* was amplified in each transgenic strain. No fragment was detected in wild-type stain 1980 (Figure 2D). The results of PCR screening demonstrated that gene *ORF I* of SsNSRV-1 was integrated into the genome of *S. sclerotiorum* strain 1980. Then the transcripts of *ORF I* were checked by RT-PCR in all the strains with primer pair (QRT-ORF1F/ QRT-ORF1R). Using cDNA as templates, a fragment of about 730 bp was detected in all positive transformants and hypovirulent strain AH98, which indicated that viral gene *ORF I* could express in all mutant strains. However, multiple fragments, which were smaller than *ORF I*, were also detected in all transformants, while only one fragment was detected in strain AH98 (Figure 2D).

### 3.3. Biological Characterization of the ORF I -Expressing Strains 

**Figure 3:**
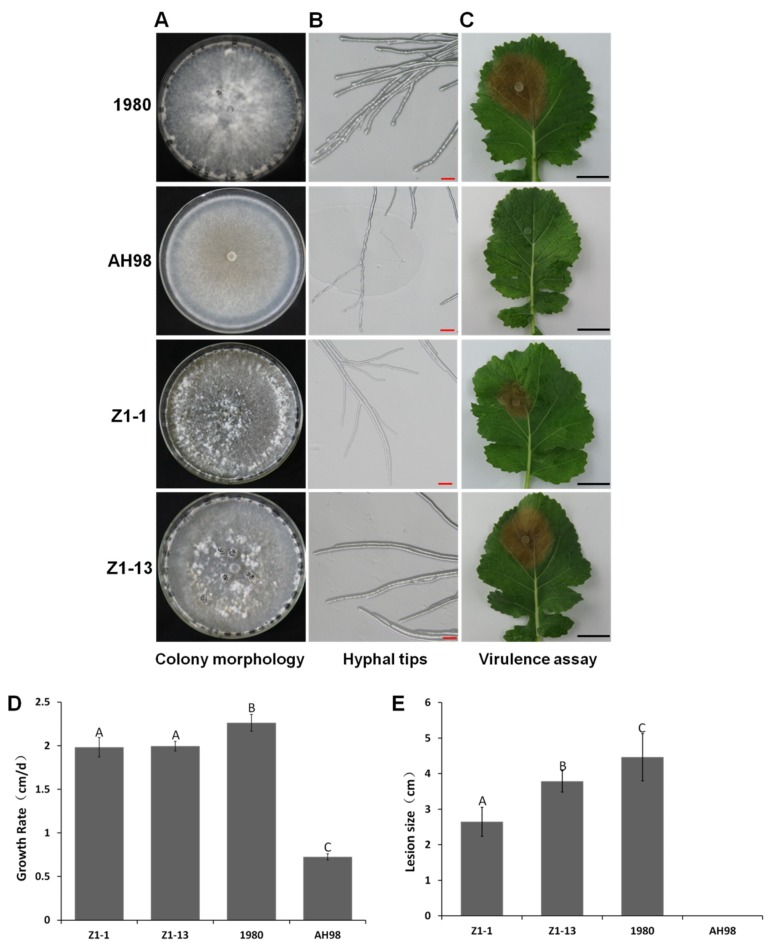
Biological characterization of Open Reading Frame Ι (*ORF I*)-expressing strains Z1-1and Z1-13. (**A**) The colonial morphology of strains Z1-1, Z1-13, 1980 and AH98 after 10 days incubation on potato dextrose agar (PDA) at 20 °C. (**B**) Hyphal tips of each strain after 48h incubation on PDA at 20 °C. The hyphal tips of strains Z1-1, Z1-13 and AH98 were narrower than that of wild-type strain 1980. (**C**) The pathogenicity of each strain after 60h incubation on the detached leaves of canola plants at 20 °C. (**D**) Growth rate of each strain on PDA at 20 °C. (**E**) The diameter of lesions induced by each strain on detached leaves of canola plants. Error bars indicate the SD from five sample means. Means followed by the different letters on the top of each column are significantly different at the *p* < 0.05 level of confidence according to Duncan’s multiple range test.

The morphology of colonies and hyphal tips of four strains, Z1-1, Z1-13, AH98, and 1980, was observed on PDA. Mutant strains Z1-1 and Z1-13 showed no significant differences from wild-type strain 1980 in colonial morphology and sclerotial formation, while the colony of strain AH98 was abnormal with more white aerial mycelia, but no sclerotium developed (Figure 3A). However, compared with wild-type strain 1980, the mutant strains had defects in morphology of hyphal tips, growth rate of mycelia and pathogenicity. The hyphal tips of strains Z1-1, Z1-13, and AH98 was narrower than that of wild-type strain 1980 (Figure 3B). The growth rate of two mutants Z1-1 and Z1-13 was about 2.0 cm/day, which was significantly lower than that of wild-type strain 1980 (2.3 cm/day) but higher than that of hypovirulent strain AH98 (0.7 cm/day) (Figure 3D). The virulence of mutant strains Z1-1 and Z1-13 was significantly reduced. After sixty hours incubation at 20 °C in humid plastic plot, two mutant strains caused much smaller lesions on detached leaves of canola than the wild-type strain 1980 did. The average diameters of lesions produced by Z1-1 and Z1-13 were 2.7 cm and 3.8 cm, while the average diameter of lesions caused by the wild-type strain 1980 was 4.5 cm. The hypovirulent strain AH98 failed to develop diseases on detached leaves of canola (Figure 3C). After genetic transformation, the exogenous fragment inserted into fungal genome only consisted of two genes, *ORF I* and a marker gene encoding hygromycin B phosphotransferase. Therefore, the phenotypic changes of two mutants Z1-1 and Z1-13 should be attributed to the expression of *ORF I* rather than that of the empty vector itself. The results demonstrated that the expression of gene *ORF I* could cause defects in vegetative growth and the pathogenicity of its fungal host.

### 3.4. Transcriptions of Viral ORFs 

Using cDNA as a template, multiple transcripts of viral *ORF I* were detected in all mutant strains by RT-PCR, which suggested that the mRNA of gene *ORF I* was alternatively spliced. To identify splicing sites, all multiple fragments were cloned and sequenced with TA-cloning. By comparing sequences of all transcripts of *ORF I,* the splicing sites of *ORF I* mRNA in mutant strains were identified as GC-AG and GT-AG. 

**Figure 4:**
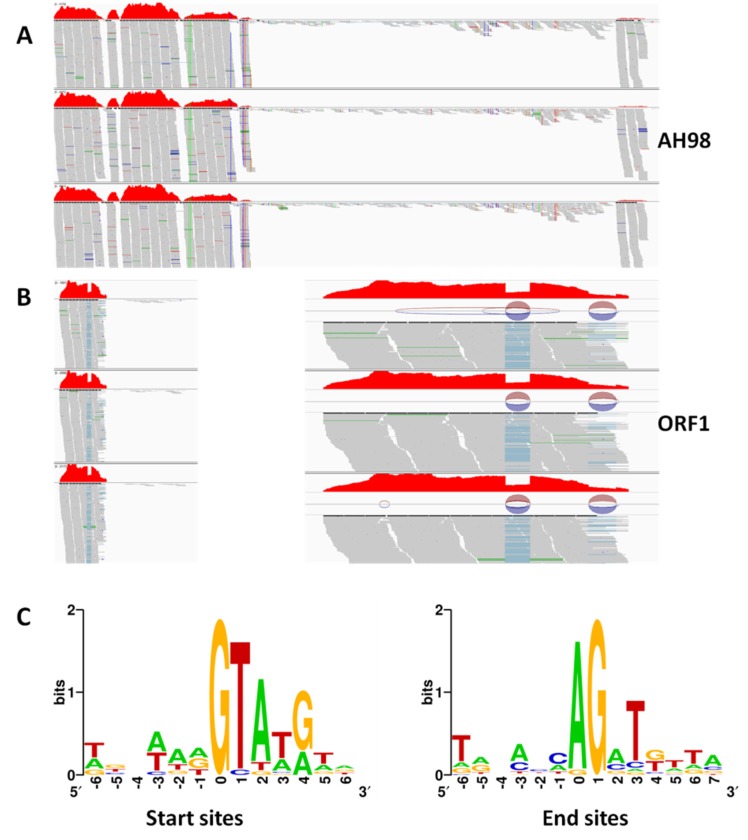
Transcriptions of viral Open Reading Frames (*ORFs*). Clean reads obtained by transcriptome analysis were mapped to SsNSRV-1 genome by software IGV (http://www.igv.org/). (**A**) The transcription of *ORF I* in strains AH98. (**B**) The sequence analysis of splicing sites of *ORF I* mRNA in the mutant strains Z1-1. The number of clean reads matching to two fragments inside *ORF I* was much less than the other regions. Multiple transcripts were detected in the mutant strain Z1-1. (**C**) Splicing fragments starts at the GT-rich sites and ends at the AG-rich sites. The blue line indicates the regions where the transcriptome data cannot match. The circles represent splice junctions.

The existence of alternative splicing of *ORF I* mRNA was also proved in the transcriptome data of mutant strains expressing *ORF I*, but not in that of hypovirulent strain AH98 (Figure 4). To verify alternative splicing in transcriptome data obtained by RNA-Seq, the clean reads were mapped to SsNSRV-1 genome. There were two splicing fragments inside gene *ORF I* (435–494, 634–701). The number of clean reads matching the two fragments was much less than the other regions of *ORF I.* The specificity of splicing sides was also confirmed in the transcriptome data. Splicing specifically recognized mRNA sequences started at the GT-rich sites and ended at the AG-rich sites (Figure 4C). 

### 3.5. Overview and Validation of Differentially Expressed Genes (DEGs)

To identify fungal gene regulated by ORF I, transcriptome sequencing was carried out between *S. sclerotiorum* wild-type strain 1980 and two *ORF I*-expressing mutant strains Z1-1 and Z1-13 using an Illumina HiSeq 2500 platform (BGI, Shenzhen, China). After sequencing, adapter sequences, ambiguous nucleotides, and low-quality raw reads were removed from original data. Over six GB Illumina clean bases were generated for each sample. On average, the genome mapping rates of reads in the data of two mutant strains Z1-1 and Z1-13, wild-type strain 1980, and SsNSRV-1 infected strain AH98 were 95.80%, 97.38%, 96.60%, and 96.73 %, respectively. The depth of sequencing was over 160× (Table S2). All the transcriptomic data were deposited in NCBI data base (Accession: PRJNA593737).

**Figure 5:**
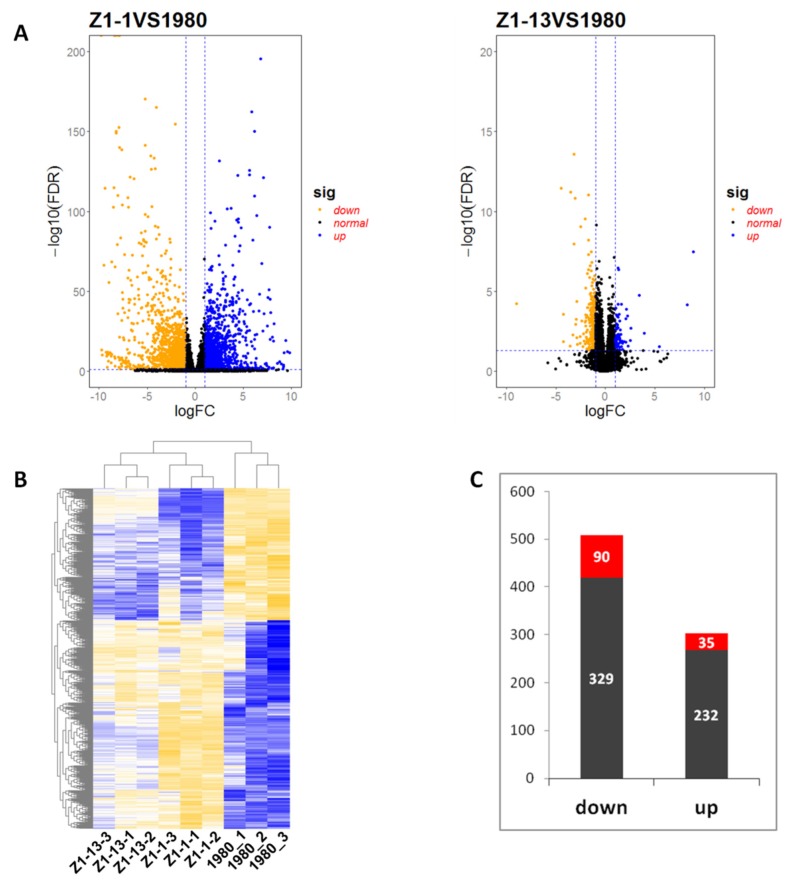
Differentially expressed genes (DEGs) between wild-type stain 1980 and Open Reading Frame Ι (*ORF I*)-expressing mutant strains Z1-1 or Z1-13. (**A**) Volcano plot displaying –log10 (FDR) on the Y-axis, index of gene differences *p*-value from NOIseq, and Log2 (fold-change) on the X-axis. The blue dots represent up-regulated DEGs between transformants and wild-type strain 1980, yellow dots represent down regulated DEGs, and black dots indicate genes without statistical significance. (**B**) Heat map of all DEGs. Row clustering was carried out based on relative expression level of genes. The column represents the individual treatment sample. Down-regulated DEGs are displayed in yellow, and up-regulated DEGs are displayed in blue. The brightness of each color corresponds to the magnitude of the difference when compared against the average value. (**C**) The number of common DEGs in mutant strains Z1-1 and Z1-13. Red columns represent DEGs with significant difference.

The results of cluster analysis indicated that three technical replicates of each strain were clustered together. DEGs were identified based on a two-fold change threshold of expression relative to wild-type strain 1980 and false discovery rate (FDR) < 0.05. The association between statistical significance and magnitude change in the dataset was also examined by volcano plot. The “volcano plot” showed the distribution of the DEGs between wild-type strain 1980 and mutant strains (Figure 5A) and a heat map represented the relative expression level of DEGs (Figure 5B). Compared with transcriptome data of strain 1980, there were 2740 up-regulated genes and 2811 down-regulated genes in the data of mutant strain Z1-1, 550 up-regulated genes and 952 down-regulated genes in the data of mutant strain Z1-13. In both mutant strains Z1-1 and Z1-13, 267 genes were commonly up-regulated, of which 35 DEGs showed significant difference, and 419 genes were commonly down-regulated, of which 90 DEGs showed significant difference (Figure 5C). All significantly differentially expressed genes are listed in Table S3.

Of these significantly DEGs, four genes (sscle_05g047430, sscle_11g081370, sscle_11g081390 and sscle_11g081490) inhibited in wild-type strain 1980 were induced in mutant strains Z1-1 and Z1-13. Among them, gene sscle_05g047430 (SS1G_05768) was a cysteinyl-tRNA synthetase participating in ATP binding, cysteinyl-tRNA aminoacylation and controlling the process of translation. Gene sscle_11g081370 (SS1G_08045) and sscle_11g081390 (SS1G_08042) were both protein kinases regulating protein phosphorylation. Gene sscle_11g081490 (SS1G_08030) was homologous to ribonucleoprotein involved in ligand-bound G protein-coupled receptor signaling pathway. There was also a gene (sscle_08g066960), which expressed in wild-type strain 1980 but was completely arrested in mutant strains Z1-1 and Z1-13. This gene contained a conserved transposon Tf2-11 domain, involved in ion binding and DNA binding. Using cellular component analysis, it was predicted to be localized in the nucleus. Sixteen annotated genes out of 35 significantly up-regulated DEGs were associated with transcription regulation, transport, metabolic process and protein modification. For example, gene sscle_09g072500 (SS1G_03912) and sscle_15g106550 (SS1G_09233) were involved in DNA binding and transcriptional regulation. Gene sscle_03g023410 (SS1G_00919) was a major facilitator superfamily (MFS) transporter, and gene sscle_05g047420 (SS1G_05770) was a hydrogen antiporter participating in the process of transmembrane transport. Three genes are involved in the metabolic process, of which gene sscle_03g022890 (SS1G_00993) was related to steroid hormone metabolic process, gene sscle_01g009260 (SS1G_01382) was supposed to encode a pigment biosynthesis protein controlling hydrolase activity, and gene sscle_08g063720 (SS1G_05048) had N-acetyltransferase activity that might control acyl-carrier-protein biosynthetic process. In addition, gene sscle_08g067700 (SS1G_05567) was related to positive regulation of GTPase activity. Gene sscle_12g088560 (SS1G_11081) was a zinc finger C6 transcription factor involved in the process of transcription regulation. Among 90 significant down-regulated DEGs, 68 were annotated, of which 36 genes were associated with pathogenesis according to Pathogen–Host Interactions database (http://www.phi-base.org/) [35], 18 genes encoded membrane proteins. For example, gene sscle_03g028700 (SS1G_00238), encoding a pectate lyase, played an important role in the virulence of *Colletotrichum gloeosporioides*. Gene sscle_04g033820 (SS1G_02334), as a glycoside hydrolase, was associated with degradation of plant cell wall during infection. Gene sscle_01g002910 (SS1G_09971) was supposed to regulate fungal virulence, as the knock-out of its homolog leaded to the loss of pathogenicity of *Cryphonectria parasitica*. The 18 genes encoding membrane proteins were mainly relevant to transmembrane transport. Among them, four genes, sscle_13g093600 (SS1G_06551), sscle_13g094450 (SS1G_06668), sscle_10g079690 (SS1G_14108) and sscle_02g015490 (SS1G_04606), were involved in ion transmembrane transport, three genes, sscle_04g038260 (SS1G_02931), sscle_02g017560 (SS1G_04336), sscle_01g006790 (SS1G_01696), were MFS-type transporter. Further, 11 genes containing DNA-binding domain were predicted to be localized in the nucleus and involved in regulation of transcription. For example, gene sscle_16g109570 (SS1G_10366), as a gata-type sexual development transcription factor, was involved in regulation of sexual sporulation by controlling formation and organization of fungal-type cell wall. Gene sscle_04g034200 (SS1G_02385) containing a C6 zinc finger domain could regulate many basal metabolisms. Furthermore, five genes, sscle_10g079690 (SS1G_14108), sscle_01g003500 (SS1G_02107), sscle_05g047540 (SS1G_05747), sscle_01g002910 (SS1G_09971), and sscle_02g013900 (SS1G_04810), were predicted to be related to mycelium development.

Taken together, ORF I could partially control host genes associated with pathogenicity, transcription, transmembrane transport, protein biosynthetic, modification, metabolic process, and so on.

### 3.6. GO Enrichment Analysis of DEGs

**Figure 6:**
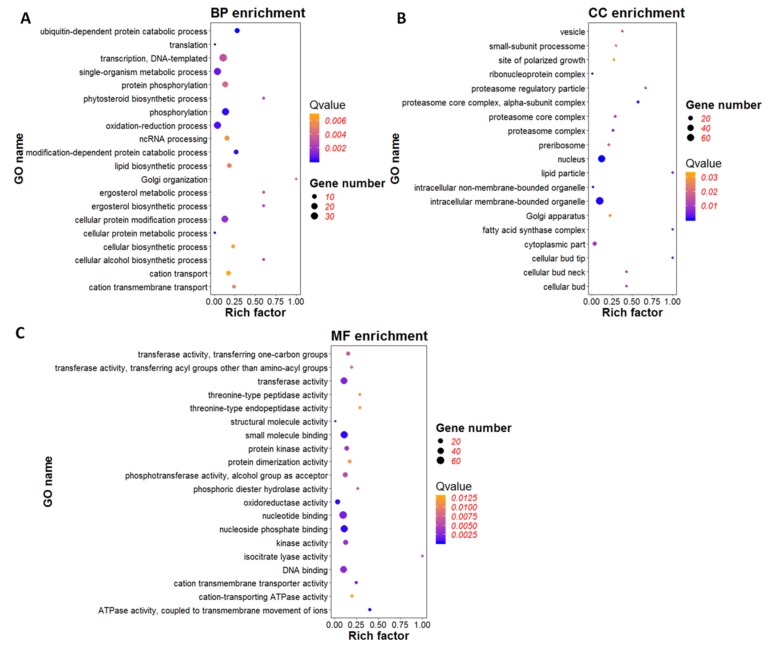
Gene ontology (GO) enrichment analysis of differentially expressed genes (DGEs). (**A**) GO enrichment analysis of DEGs in biological process (BP). (**B**) GO enrichment analysis of DEGs in cellular component (CC). (**C**) GO enrichment analysis of DEGs in molecular function (MF). The bubble graph displays GO items on the Y-axis and rich factor on the X-axis. The color of bubbles represents Q value. The size of bubbles represents the number of genes.

To determine significant gene ontology (GO), GO enrichment analysis of 686 DEGs was performed using *p*-value < 0.05 as a threshold. The GO items used in this study including three major functional ontologies, biological process, cellular component, and molecular function [32]. DEGs associated with the biological process were mainly involved in ubiquitin-dependent protein catabolic process (GO: 0006511), modification-dependent protein catabolic process (GO: 0019941), phosphorylation (GO: 0016310) and oxidation-reduction process (GO: 0055114) (Figure 6A). DEGs related to cellular components were mainly classified into nucleus (GO: 0005634), intracellular membrane-bounded organelle (GO: 0043231), proteasome core complex, and alpha-subunit complex (GO: 0019773) (Figure 6B). The results indicated that genes might be involved in the regulation of nucleus, protein synthesis and modification, and intracellular membrane composition were regulated by the expression of viral gene *ORF I*. In the category of molecular function, DGEs were primarily involved in binding and activation, including nucleoside phosphate binding (GO: 1901265), small molecule binding (GO: 0036094), nucleotide binding (GO: 0000166), DNA binding (GO: 0003677), ATP binding (GO: 0005524), ATPase activity, coupled to transmembrane movement of ions (GO: 0042625), oxidoreductase activity (GO: 0016491), transferase activity (GO: 0016740), and kinase activity (GO: 0016301) (Figure 6C). All of these were the basic functions of life activities. 

The result of GO enrichment analysis indicated that viral gene *ORF I* might regulate transcription, translation and modification of proteins. In addition, intracellular membrane composition might also be regulated to enhance the activity of transmembrane transporter to facilitate the invasion and replication of the virus.

### 3.7. KEGG Pathway Analysis of DEGs

**Figure 7:**
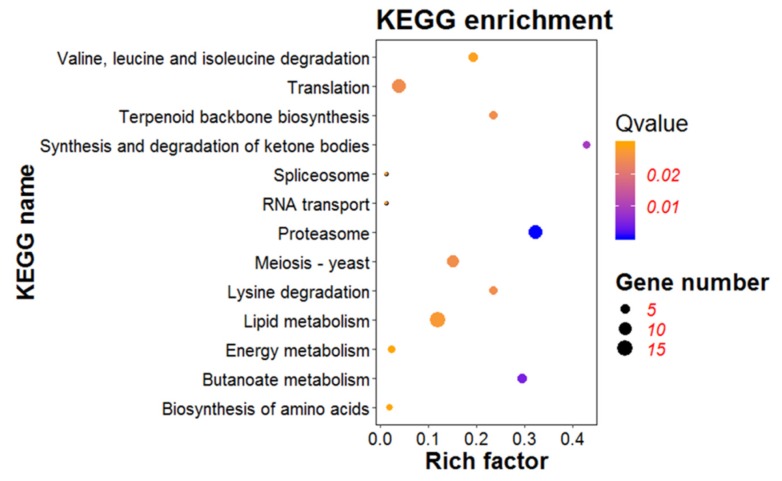
Kyoto encyclopedia of genes and genomes (KEGG) enrichment analysis of differentially expressed genes (DEGs). The bubble graph displays KEGG items on the Y-axis and rich factor on the X-axis. The size of bubbles represents the number of genes. The color of bubbles represents Qvalue.

KEGG pathway analysis was conducted to determine specific pathways induced or suppressed by the expression of *ORF I* [33]. FungiFun was applied for pathway enrichment analysis. The results indicated that 12 KEGG pathways were extracted based on 686 DEGs (Figure 7). Of them, proteasome (ko03050), butanoate metabolism (ko00650), synthesis and degradation of ketone bodies (ko00072) were the three mainly enriched pathways, which might be involved in response to *ORF I*. The pathways of amino acid biosynthesis and metabolism were also identified, including lysine degradation (ko00310), valine, leucine, and isoleucine degradation (ko00280), and biosynthesis of amino acids (ko01230). Meiosis–yeast pathway (ko04113) related to the hyphal growth and development was also enriched. At the same time, the pathway of gene transcription and translation were mapped as translation (ko22000), spliceosome (ko03040), RNA transport (ko03013). In addition, two important metabolic pathways, namely lipid metabolism (ko14000) and energy metabolism (ko13000), were also changed in the mutant strains Z1-1 and Z1-13. Thus, as a viral gene, *ORF I* might regulate the transcription and translation of certain genes of the host, and also disrupt the metabolic balance of amino acid and lipid in the host.

### 3.8. Heat Map Enrichment Analysis of DEGs Associated with Secretory Proteins and Pathogenesis

**Figure 8:**
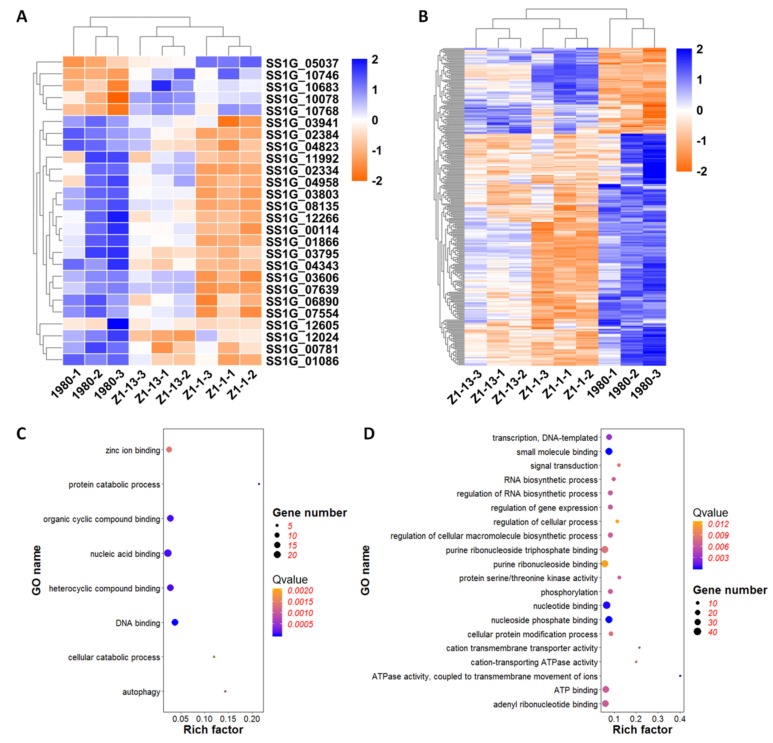
Heat map enrichment analysis of differentially expressed genes (DEGs) associated with secretory proteins and pathogenicity. (**A**) Heat map enrichment analysis of DEGs encoding putative secretory proteins. (**B**) Heat map enrichment analysis of DEGs related to fungal pathogenesis. (**C**) GO enrichment analysis of up-regulated DEGs associated with pathogenicity. (**D**) GO enrichment analysis of down-regulated DEGs associated with pathogenicity. The heat map displays gene names on the Y-axis and sample names on the X-axis. The color represents expression level of genes.

We predicted secretory proteins in the genome of *S. sclerotiorum* according to the secrete proteins prediction method [36]. Of 686 DEGs, 26 genes were predicted to encode secrete proteins, including five up-regulated genes and 21 down-regulated genes (Figure 8A). The five up-regulated genes mainly involved in oxidation-reduction process and metabolic process. Of them, gene SS1G_05037 encoded a flavin mononucleotide-binding split barrel-related protein related to electron transfer during breathing and other biological oxidation process, gene SS1G_10078 had peroxidase activity domain which might respond to oxidative stress, gene SS1G_10683 encoded a carbohydrate binding protein in hexose metabolic process. Gene SS1G_10768 was a variant-surface-glycoprotein phospholipase C related to lipid metabolic process, while SS1G_10746 was similar to clathrin light chain protein, as the major protein of the polyhedral coat of coated pits and vesicles, involved in the stabilization of kinetochore fibers of the mitotic spindle. Of the down-regulated genes, eight genes with hydrolase activity domain were associated with the carbohydrate metabolic process. For example, gene SS1G_02334 encoded a glycoside hydrolase in extracellular region and participated in glucan catabolic process. Gene SS1G_03606 was annotated as a glucan-beta-glucosidase that involved in starch and sucrose metabolic process. Gene SS1G_12605, encoding a subtilisin-like protein with serine-type endopeptidase activity, could active evasion of host immune response via regulation of host complement system to effect pathogenesis. Of the down-regulated genes, six genes were relevant to protein modification and proteolysis. Of the six genes, SS1G_00114 encoded a nucleoside diphosphatase targeting Golgi membrane and involved in cellular protein modification process. SS1G_03941 had aspartic-type endopeptidase activity domain and pertinented to proteolysis. In addition, SS1G_00781 was a transcription factor to regulate transcription and SS1G_03795 was a ribonuclease T2 that effected RNA binding.

Among the 686 DEGs, 284 genes were associated with pathogenesis according to Pathogen-Host Interactions database (http://www.phi-base.org/) [35], of which 77 genes were up-regulated and 207 were down-regulated (Figure 8B). GO enrichment analysis indicated that the up-regulated genes were mainly involved in DNA binding (GO: 0003677), nucleic acid binding (GO: 0003676), zinc ion binding (GO: 0008270), heterocyclic compound binding (GO: 1901363), and organic cyclic compound binding (GO: 0097159). Many up-regulated genes encoded DNA repair protein (SS1G_00096, SS1G_03059 and SS1G_03060) and zinc finger protein (SS1G_05816, SS1G_07778, SS1G_11081) that regulated transcription. Further, SS1G_04900 was an isocitrate lyase that played an important role in glyoxylate cycle. GO enrichment analysis of the down-regulated genes indicated that they were primarily involved in small molecule binding (GO: 0036094), nucleotide binding (GO: 0000166), ATP binding (GO: 0005524), phosphorylation (GO: 0016310), regulation of gene expression (GO: 0010468), signal transduction (GO: 0007165), transferase activity (GO: 0016740), kinase activity (GO: 0016301), which were closely related to the growth, development, and pathogenicity process.

The enrichment analysis of DEGs associated with secretory proteins and pathogenicity demonstrated that viral gene *ORF I* might regulate the transcription, translation, and post-translation processing through the function of DNA binding, nucleotide binding, signal transduction, and phosphorylation. At the same time, the genes involved in ATP binding and many kinase activity which affected basic functions of life activities were also enriched.

## 4. Discussion

In this study, we predicted protein secondary structure of *ORF I* of SsNSRV-1 and integrated the gene into the genome of *S. sclerotiorum* strain 1980 to perform functional analysis. Fungal genes responding to *ORF I* -expression were also investigated by transcriptome analysis.

From transcriptome data, 26 predicted secretory proteins were found to be regulated by ORF I significantly, of which eight down-regulated secretory proteins had hydrolase activity and might influent plant cell wall digestion during fungal infection process. In addition, 41.4 % (284 DEGs) of 686 significantly DEGs were demonstrated to be associated with pathogenesis according to Pathogen-Host Interactions database [35]. Many DEGs encoded zinc finger proteins that could regulate the expression of genes as transcription factor. Some zinc finger proteins were demonstrated to regulate the growth, development, metabolism, and pathogenesis of pathogenic fungi. For example, a novel Zn_2_Cys_6_ transcription factor BcGaaR could regulate D-galacturonic acid utilization in *Botrytis cinerea* [37]. A unique Zn(II)_2_-Cys_6_-type protein, KpeA, was involved in secondary metabolism and conidiation in *Aspergillus oryzae* [38]. A functionally conserved Zn_2_Cys_6_ transcription factor regulated necrotrophic effector gene expression and host-specific virulence of two major *Pleosporales* fungal pathogens of wheat [39]. We also found several genes predicted to be associated with mycelium development, such as sscle_10g079690 (SS1G_14108), sscle_01g003500 (SS1G_02107), sscle_05g047540 (SS1G_05747), sscle_01g002910 (SS1G_09971), sscle_02g013900 (SS1G_04810). And genes SS1G_00204, SS1G_01065, SS1G_03818 might play a crucial role in cell morphogenesis. These DEGs indicated that *ORF I* regulated the genes relevant to cell morphogenesis and pathogenesis. Thus, the defects in the vegetative growth and virulence of *S. sclerotiorum* strains infected by SsNSRV-1 maybe attributed to the expression of viral gene *ORF I*.

Interestingly, multiple transcripts of viral gene were detected in every mutant strain expressing *ORF I* but not in the hypovirulent strain AH98 carrying SsNSRV-1. The intron-exon boundaries which contains the typical motif for eukaryotic spliceosomal intron-splice donor/acceptor sites (GT-AG) were identified by analysis the sequences of mRNA splice sites [40]. Thus, the intron cleavage system of the fungal host could perform post-transcriptional processing of the mRNA of the viral gene *ORF I*. The integrity of the SsNSRV-1 genome might protect its own genes from splicing by the host intron cleavage system. As is well known, alternative RNA splicing was intensively performed by eukaryotes to increase proteome diversity through the formation of numerous mRNA isoforms from a primary transcript and to regulate the expression of proteins in different organs and cell types [22,23]. It was also an important mechanism that had been reported to be used by eukaryotic hosts to regulate their defense systems at the cellular level [20,24,28]. In our transcriptome data, a large number of genes related to the alternative splicing, such as serine-arginine (SR) proteins, ribonucleoprotein, spliceosome, proteasome, were enriched [28,41,42]. It seemed that the fungal host *S. sclerotiorum* performed post transcriptional processing for the mRNA of *ORF I* by an alternative splicing mechanism.

At the same time, some researches had demonstrated that viral infection might affect the patterns of mRNA splicing in host cell. Many viruses had been reported to take use of this strategy to express their own proteins during the infection cycle. Most of them were animal viruses, complex retroviruses and type A influenza virus [24,28]. Although splicing was rarely observed in plant viruses, the splicing of viral mRNA was detected in several plant DNA viruses in two families *Geminiviridae* and *Caulimoviridae* [29,30]. The splicing of intron was known to occur in the nucleus and catalyzed by the spliceosome, a large and highly dynamic ribonucleoprotein complex [28,42,43,44]. However, the manipulation of the host splicing machinery was not only limited in nuclear viruses, but also observed in viruses that replicated in the cytoplasm, such as picornaviruses and flaviviruses [28,45]. This could be accounted for the nucleocytoplasmic shuttling of some viral proteins (e.g., the dengue virus NS5 protein [43]) and splicing factors (e.g., SR proteins [41]), increased nuclear permeability upon viral infection [46], and signaling pathways triggered by viral infection [47]. As mentioned above, a nuclear localization signal was predicted in ORF I, so it was possible for ORF I to shuttle between the nucleus and the cytoplasm of host cell and bind with host spliceosome to implement alternative splicing in mutant strains. A large number of genes closely related to the alternative splicing, such as ribonucleoprotein, spliceosome, proteasome, were reprogramed by ORF I in the transcriptome data. Those results demonstrated that alternative splicing might be a key molecular mechanism in SsNSRV-1–*S. sclerotiorum* interaction. In addition, SsNSRV-1 was a negative-strand RNA virus whose transcription happened in the cytoplasm rather than in the nucleus where the splicing machine was located. Therefore, the alternative splicing of viral mRNA might be an artificial phenomenon due to the expression of *ORF I* as a nuclear gene. This possibility needed to be verified in future. We could mutate the splicing signal in *ORF I* without changing the open reading frame and then express it in *S. sclerotiorum*.

Order *Mononegavirales* consists of five families, namely *Bornaviridae*, *Filoviridae*, *Paramyxoviridae*, *Rhabdoviridae,* and *Nyamiviridae*. All viruses in this order were characterized as a single long non-segmented RNA genome and shared similar genomic structure: N-P-G-M-L [48]. In a canonical virus NP structural family of NSRV, N- and C-lobes were mainly formed by a set of α-helices. N- and C-extensions were constituted by loop and/or α-helix [49,50]. In previous study, ORF II was proved as a N protein without significant similarity with other viruses in *Mononegavirales* by SDS-PAGE protein electrophoresis analysis. Using peptide mass fingerprinting analysis, *ORF I*-encoded peptides were also identified in the virion crude extract of SsNSRV-1. The prediction of secondary structure demonstrated that ORF I contained many helix, one coiled coil, two DNA-binding sites (76–83, 227–237), and one protein binding region [50]. The morphology of the particles of SsNSRV-1 was previously observed by transmission electron microscopy (TEM): filamentous, 25–50 nm in diameter, ~1000 nm in length. When the particles were broken, helical and flexible substructures were observed. The nucleocapsid of SsNSRV-1 was a single left-handed helical structure with a diameter of 20–22 nm and a length of 200–2000 nm when tightly coiled [8]. Therefore, we speculated that ORF I might be involved in the formation of viral particles as the support structure of nucleocapsids.

G protein-coupled receptors (GPCRs), belonging to a large transmembrane receptor superfamily, was involved in many cellular signaling pathways in the cell. Small G proteins with a molecular weight of 20–30 KDa could independently function as a hydrolase that binds and hydrolyzes inactive guanosine triphosphate (GTP) to form active guanosine diphosphate (GDP) [51]. It was demonstrated that viruses could interfere with small G protein signal transduction pathways to enhance their replication in both plants and animal hosts. For example, the oncogenic Kaposi’s sarcoma-associated herpesvirus (KSHV), human herpesvirus 8, contained a constitutively active GPCR homolog, which could stimulate the secretion of vascular endothelial growth factor (VEGF), a key angiogenic stimulator and a critical mitogen for the development of Kaposi’s sarcoma [52]. Human herpesvirus 6 (HHV-6), a ubiquitous T-lymphotropic beta-herpesvirus, encoded two GPCR homologs (U12 and U51), of which U51, as a positive regulator of viral replication in vitro, could promote membrane fusion and facilitates cell-cell spread of this highly cell-associated virus [53]. At the same time, host’s heterotrimeric G-proteins were also reported to play a positive role in defense against viral pathogens Cucumber mosaic virus (CMV) (*Bromoviridae*) and Turnip mosaic virus (TuMV) (*Potyviridae*) [54]. In this study, 4 fungal genes related to G protein signal transduction were significantly differentially expressed in the mutant strains Z1-1 and Z1-13. Of four genes, sscle_09g068890 (SS1G_10918) participated in regulation of small signal transduction mediated by GTPase, the other three genes were GPCRs. The expression of viral ORF I interfered with G protein signal transduction pathways of *S. sclerotiorum*. However, we didn’t know if this effect was to facilitate the infection and replication of SsNSRV-1 in the host or to trigger the defense of host. 

In addition, GO and KEGG enrichment analysis indicated that many genes related to nucleotide, DNA and RNA binding, transcription, transmembrane transporter and protein synthesis and modification were regulated by ORF I. ORF I mutant strains showed narrow hyphae tips, lower growth rate and hypovirulence compared with wild-type strain 1980. Taken together, we inferred that viral ORF I could regulate the transcription, translation, and modification of host genes to facilitate the proliferation of itself and reduce the virulence of host. Therefore, *ORF I* may be a potential gene used for prevention of *S. sclerotiorum*. 

But, how SsNSRV-1 modified the host and conferred hypovirulence to fungi required more research. Although the prediction of secondary structure indicated that ORF I had a nuclear localization signal and two DNA binding sites, there was no direct evidence for the cellular localization of this protein. Further study need to confirm if ORF I can target the nucleus of host to reprogram the host gene expression. At the same time, since there was another hypovirus SsHV-1 in strain AH98, we did not compare the transcriptome data of the AH98 strain with those of other strains. In future studies, we will use strains infected only with SsNSRV-1 as a positive control for transcriptome analysis, which may provide more valuable information for understanding the function of ORF I in facilitate viral proliferation and reduce the virulence of host. It should also be noted that *ORF I* might not be the only gene associated with hypovirulence in the SsNSRV-1 genome. Our preliminary research on other ORFs implied that there might be another viral ORF, whose expression could reduce the pathogenicity of *S. sclerotiorum* [55].

## Figures and Tables

**Table 1:** Predicted function of protein Open Reading Frame Ι (ORF I).

Hit Name	Function	Probability	E-Value
5CWS_D	Nucleoporin; nucleocytoplasmic transport, PROTEIN TRANSPORT	82.52	8.3
3TNU_B	Keratin, type I cytoskeletal 14; Coiled-coil, Structural Support	71.8	22
4EGW_A	Magnesium transport protein CorA; magnesium transporter, magnesium binding	68.46	48
5IJO_S	Nuclear pore complex protein Nup155; Nuclear pore complex, Nucleocytoplasmic transport	66.44	23
1NLW_E	MAD PROTEIN/MAX PROTEIN/DNA; transcription factor	64.78	8.7
1NLW_A	MAD PROTEIN/MAX PROTEIN/DNA; transcription factor	64.72	11
4ZRY_A	Keratin, type I cytoskeletal 10; keratin, intermediate filament, coiled-coil	63.03	50
3NVO_A	Zinc transport protein zntB	61.26	90
5N77_C	Magnesium transport protein CorA; Homopentamer Complex Transport Membrane, transport	60.49	87
6GAO_B	Outer capsid protein sigma-1; cell attachment protein, reovirus sigma1	57.97	74
2O3E_A	Neurolysin, thermolysin-like domain, substrate-binding channel	52.51	110
4RSI_B	Structural maintenance of chromosomes protein; Smc hinge domain with coiled	52.12	140
6G1L_A	Microphthalmia-associated transcription factor/DNA Complex; melanocyte, autophagy, transcription factor	51.9	40
5IJO_H	Nuclear pore complex protein Nup155, Nucleocytoplasmic transport	50.41	120
6GAP_A	Outer capsid protein sigma-1; cell attachment protein, reovirus sigma1	50.13	93
4ATH_A	DNA binding protein, transcription factor	50.13	96

## Supplementary Materials

The following DATA are available online at https://www.mdpi.com/1999-4915/12/4/456/s1, Table S1: Primers used in this study; Table S2: Summary of sequencing data; Table S3: Significantly differentially expressed genes in transcriptome data.
