# Supplementary material for: ORF Ι of Mycovirus SsNSRV-1 is Associated with Debilitating Symptoms of Sclerotinia sclerotiorum
Source: Viruses. 2020 Apr 17;12(4):456. doi: 10.3390/v12040456 (PMC7232168; doi:10.3390/v12040456)
Supplement: Supplementary file 1 [file viruses-12-00456-s001.zip › viruses-766358.suppl zip/Supplementary Files/TableS1.pdf]

Table S1 Primers used in this study

Primer Name	Sequences（5'-3'）
ORF1F	GGGGTACCATGAGCACAACTGTTGACATTAAGA
ORF1R	GGGGTACCCTACTTTCTGAGATGCTTCGGAAGC
QRT-ORF1F	GCCACTCGTCTGTGTCCCT
QRT-ORF1R	GTTGAGTTAGTAGTGATTTTTTGCA
SsActinF1	AGCACCAGAGGAGCACCCAGTTT
SsActinR1	CGTGAAGATTGACTGGCGGTTTG
SSActinF2	CCAATCAACCCAAAGTCCAACAG
SSActinR2	CGAGCAATGGCGTGAGGAAGTG
EFTF	GCAACTCTCCTTCTTCCTTCTTCG
Ptrp-R	CTGGACGACTAAACCAAAATAGCA
