# Supplementary material for: ORF Ι of Mycovirus SsNSRV-1 is Associated with Debilitating Symptoms of Sclerotinia sclerotiorum
Source: Viruses. 2020 Apr 17;12(4):456. doi: 10.3390/v12040456 (PMC7232168; doi:10.3390/v12040456)
Supplement: Supplementary file 1 [file viruses-12-00456-s001.zip › viruses-766358.suppl zip/Supplementary Files/TableS2.pdf]

Table S2 Summary of sequencing data

Sample Name	Clean Reads	Clean bases	Clean bases (GB)	Map quality (%)	Q30 (%)	Q20 (%)	GC (%)	Sequencing Depth (X)
Z1-1-1	41,396,834	6,209,525,100	6.21	95.59	92.99%	97.31%	46.64%	161
Z1-1-2	41,032,438	6,154,865,700	6.15	95.80	93.07%	97.34%	46.61%	160
Z1-1-3	41,570,024	6,235,503,600	6.24	96.02	92.96%	97.29%	46.61%	162
Z1-13-1	41,287,986	6,193,197,900	6.19	97.04	92.96%	97.29%	46.43%	161
Z1-13-2	41,847,698	6,277,154,700	6.28	97.25	93.22%	97.40%	46.39%	163
Z1-13-3	40,812,668	6,121,900,200	6.12	97.86	92.83%	97.22%	46.22%	159
1980-1	41,574,046	6,236,106,900	6.24	98.07	93.79%	97.61%	46.33%	162
1980-2	41,688,260	6,253,239,000	6.25	97.15	93.80%	97.62%	46.47%	163

1980-3	42,077,430	6,311,614,500	6.31	97.59	93.57%	97.52%	46.54%	164
AH98-1	41,910,418	6,286,562,700	6.29	96.11	93.97%	97.70%	46.57%	163
AH98-2	41,119,348	6,167,902,200	6.17	97.09	93.97%	97.72%	46.70%	160
AH98-3	40,973,600	6,146,040,000	6.15	97.00	93.84%	97.66%	46.66%	160

Z1-1-1, Z1-1-2 and Z1-1-3: Three technical replicates of mutant strain Z1-1 expressing *ORF I*. Z1-13-1, Z1-13-2 and Z1-13-3: Three technical replicates of another mutant strain Z1-13 expressing *ORF I*. 1980-1, 1980-2 and 1980-3: Three technical replicates of wild-type strain 1980. AH98-1, AH98-2, AH98-3: Three technical replicates of hypovirulent strain AH98. Map quality: Genome mapping reads/clean reads. Q30 (%): Percentages of clean bases whose correct base recognition rates are greater than 99.9% in total bases. Q20 (%): Percentages of clean bases whose correct base recognition rates are greater than 99% in total bases. Sequencing depth: Clean bases/genome size.
